# Factors associated with initiation of pharmacological therapy and treatment changes in postural orthostatic tachycardia syndrome

**DOI:** 10.3389/fneur.2024.1411960

**Published:** 2024-06-20

**Authors:** Samantha Jean Stallkamp Tidd, Ryan X. Zhang, Christopher Cantrell, Amy S. Nowacki, Tamanna Singh, Robert Wilson

**Affiliations:** ^1^Cleveland Clinic Lerner College of Medicine, Cleveland, OH, United States; ^2^Keck School of Medicine, University of Southern California, Los Angeles, CA, United States; ^3^Department of Quantitative Health Sciences, Cleveland Clinic Foundation, Cleveland, OH, United States; ^4^Department of Cardiovascular Medicine, Heart, Vascular, and Thoracic Institute, Cleveland Clinic Foundation, Cleveland, OH, United States; ^5^Department of Neuromuscular Medicine, Cleveland Clinic Foundation, Cleveland, OH, United States

**Keywords:** POTS, treatment, migraines, fatigue, heart rate, neck pain, management

## Abstract

**Purpose:**

Postural Orthostatic Tachycardia Syndrome (POTS) is a heterogenous disorder of the autonomic nervous system that is often disabling. There are no FDA-approved therapies for the treatment of this condition. While some patients recover with baseline non-pharmacological intervention, others require repeated trials of off-label pharmacological therapies. The reasoning for this variable treatment response is unknown. The purpose of this work is to identify potential factors that are associated with higher odds of starting pharmacotherapy and/or a higher rate of POTS treatment changes.

**Methods:**

Chart review of demographic, disease and treatment descriptions, medical history, and tilt table examinations of 322 POTS patients who were diagnosed between 2018 and 2020 at our tertiary care center was completed. We first identified the most significant factors associated with an increased odds of starting pharmacotherapy using variable selection techniques and logistic regression. We then identified the most significant factors associated with changes in POTS treatment strategies using variable selection techniques and negative binomial regression modeling. A significance level of 0.05 was utilized.

**Results:**

A total of 752 POTS-specific treatment courses were cataloged, and 429 treatment changes were observed. The most cited reason for a change in management was uncontrolled symptoms. History of migraine headaches, reported fatigue, reported palpitations and a previous POTS diagnosis at an outside institution were found to be associated with a higher odds of starting pharmacotherapy for POTS symptoms (Odds Ratio of 2.40, 1.94, 2.62, 2.08, respectively). History of migraine headaches, reported fatigue, and higher heart rate differences on tilt table examination were found to be associated with an increase in the rate of POTS treatment changes (44, 66, 13% increase in incidence rate, respectively), while reported neck pain was associated with a decrease (27% decrease in incidence rate).

**Conclusion:**

Our work identifies important areas of focus in the development of high-quality trials involving both the non-pharmacological and pharmacological treatment of POTS and highlights several characteristics of patients that may be more refractory to both baseline non-pharmacological treatments and current pharmacological treatment strategies.

## 1 Introduction

Postural Orthostatic Tachycardia Syndrome (POTS) is a heterogeneous, often debilitating disorder of the autonomic nervous system. The syndrome is largely characterized by chronic orthostatic intolerance with an excessive increase in heart rate upon standing (1, 2). Other than orthostatic intolerance, patients experience fatigue, headaches, cognitive impairment, chest pain, and gastrointestinal symptoms (1). These symptoms are often disabling for patients, leading to decreased quality of life, ability to work, and income loss (3, 4).

While the true prevalence of POTS is unknown due to the lack of widespread epidemiological studies of the condition as well as rampant misdiagnosis, it is estimated to affect between 0.1 and 1% of the US population (5). The number of patients with this condition in the United States is growing due to the recent onslaught of individuals developing POTS after COVID-19 infection (6). Given the increasing population affected by this disorder, and the significant disability that comes with it, there is a large need for research surrounding the treatment of these patients (3, 6).

First-line therapy for POTS is non-pharmacological treatment with increased salt intake and hydration, compression garments, and exercise. However, many POTS patients require additional therapies (7). There are no universally successful therapies for POTS, and most patients require a combination of pharmacological treatment, lifestyle changes, and physical therapy (1, 8). There are no FDA-approved therapies for POTS, relatively few high-quality randomized controlled clinical trials on the pharmacological treatments, and no standardized treatment algorithms to guide therapy (1, 8). Clinicians largely follow their own clinical judgment and the loose recommendations of national societies that are not tailored to individual patients when choosing pharmacological therapies in addition to baseline non-pharmacological management. While some patients experience symptom resolution with very little intervention, others require trials of many different therapies with frequent exacerbations of symptoms (1, 8). There are no studies that have found ways to identify patients whose symptoms are more likely to be refractory to current treatment approaches, or those that need pharmacological therapy in the first place (9). These “treatment-refractory” patients may need closer follow-up, alternative treatment approaches, or may represent a subgroup of POTS patients that needs further study. The first aim of this study is to identify patient-level factors that may be associated with the need for pharmacological therapy in addition to baseline non-pharmacological management. Our second aim is to identify patient-level factors associated with repeated treatment strategy changes in our autonomic clinic in a large tertiary care center.

## 2 Materials and methods

### 2.1 Chart review

In this retrospective, institutional review board-approved cohort study, the electronic medical records of 322 patients initially seen at a tertiary academic medical center between 2018 and 2020 with confirmed POTS diagnoses via tilt table testing were extrapolated. Demographic data, social history, past medical history, medications, and tilt table testing were collected from the closest available electronic medical record entry within 3 months of the first visit with a POTS specialist at our institution. Reported symptoms at the first visit were additionally recorded. Supplementary Table S1 lists each collected variable used and their definitions.

#### 2.1.1 Aim 1: identifying factors associated with the initiation of pharmacological therapy in POTS

The primary outcome measure for aim 1, the initiation of pharmacological therapy, was collected via a review of patients’ charts. Descriptive statistics of the population are reported as either percent (count), minimum and maximum, mean (SD), or median (interquartile range). All descriptive statistics were acquired utilizing JMP Pro version 16 (SAS Institute Inc., Cary, NC, 1989–2021).

#### 2.1.2 Aim 2: identifying factors associated with a higher rate of treatment strategy changes in POTS

In line with several other studies focused on quality of care, treatment change was selected as our outcome (10–13). A treatment course change was defined as a change or discontinuation of a POTS-specific treatment regimen after an initial visit with a POTS specialist at our institution. Dose adjustments were not considered management changes. A planned treatment course completion followed by a wean was not considered a change. All patients at our institution received baseline non-pharmacological management in addition to any pharmacological management course. Non-pharmacological management includes counseling on salt, fluids, compression garments and exercise. The addition or subtraction of medications beyond these universal recommendations were considered treatment changes.

**Figure 1:**
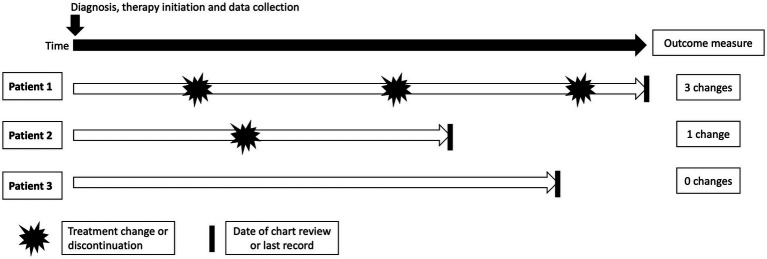
Chart review timeline. A visual depiction of the data collection process of three patients. Predictor variable collection occurred at the arrow, time zero. Treatment changes (represented by the pointed shape) were collected from time zero until the date of chart review or last record (represented by the solid black line).

The primary outcome measure, the number of treatment course changes, was collected via a review of patients’ charts. The timeframe of the notes reviewed was additionally recorded, as well as the charted reasons for treatment change. A pictorial representation of the timeline of data collection can be seen in Figure 1.

### 2.2 Inclusion and exclusion criteria

Patients were seen at the Cleveland Clinic for an initial visit between 2018 and 2020 in the Neuromuscular department, underwent testing and clinical evaluation, and meet the criteria for POTS by tilt table testing:

1. Sustained elevation in heart rate of at least 30 beats per minute within the first 10 min of upright tilt for adults over 19. An increase of at least 40 beats per minute is required for those less than 19.
2. Absence of sustained orthostatic hypotension (a decrease in systolic blood pressure of at least 20 mmHg or a decrease in diastolic blood pressure of at least 10 mmHg) between 3 to 10 min of upright tilt.
3. Symptoms of orthostatic intolerance for at least 3 months by the time of the initial visit.
4. Diagnosis confirmed by a physician.
5. Other causes for tachycardia or orthostatic intolerance have been eliminated (for example, atrioventricular nodal reentrant tachycardia or Multiple Systems Atrophy).

Patients that did not meet the inclusion criteria by any one factor were excluded from analysis.

### 2.3 Variable selection

Through extensive chart review described above, numerous demographic and clinical variables were collected. Only those variables without missing data were utilized in the study and are listed in Supplementary Table S1. All study variables could not reasonably be placed within one model for each aim without overfitting. Thus, to determine the subset of variables to include in our models, recognizing that several variable selection methods exist (each with their own strengths and weaknesses), we utilized 6 methods of variable selection for each aim (14).

Forward selection is a variable selection method that starts with no variables and adds variables to the model one at a time. If a variable is entered into the model, the variable will remain and cannot be removed. We used two methods for determining which variable was selected at each step with forward selection, a smallest *p*-value (<0.05) approach and the AIC (Akaike’s information criterion, an estimator of predictive accuracy (15)). Backward elimination is a variable selection method that starts with all variables in the model and progressively removes variables. If a variable is removed from the model, the variable will remain and cannot be reentered. We again used two methods for determining which variable was removed at each step with backward selection, a largest *p*-value (>0.05) and AIC (Akaike’s information criterion). Stepwise selection is similar to forward selection, except variables can be removed if later found to be insignificant when other variables are added. An entry/exit criterion of 0.05 was utilized. Finally, we implemented LASSO, a supervised algorithm wherein the process identifies those variables most strongly associated with the outcome (variable selection) and then based on a penalty, forces the coefficients of the weakest variables toward zero (shrinkage) (14).

Supplementary Table S1 lists all candidate variables. Tables 1, 2 show which variables were selected by each of the six different methods (indicated with a checkmark) as well as how many times a variable was selected for each aim. All covariates were evaluated for multicollinearity before their inclusion via variance inflation factors (16). SAS statistical software version 9.4 (SAS Institute, Cary, NC) and JMP Pro version 16 (SAS Institute Inc., Cary, NC, 1989–2021) were utilized for variable selection methods.

**Table 1:** Variable selection results for aim 1 pharmacological treatment initiation: results of each variable selection method and the number of times each variable was selected.

Variable	Forward selection 0.05	Forward selection AIC	Backwards elimination 0.05	Backwards elimination AIC	Stepwise	LASSO	Times selected
Sex		✓					1
History of migraine*	✓	✓	✓	✓	✓	✓	6
Palpitations*	✓	✓	✓	✓	✓	✓	6
Fatigue*	✓	✓	✓	✓	✓		5
Previously diagnosed?*	✓	✓	✓	✓	✓	✓	6

*Indicates variable was ultimately selected for the model.

**Table 2:** Variable selection results for aim 2 treatment changes: results of each variable selection method and the number of times each variable was selected.

Variable	Forward selection 0.05	Forward selection AIC	Backwards elimination 0.05	Backwards elimination AIC	Stepwise	LASSO	Times selected
Age						✓	1
Department						✓	1
History of autoimmune condition				✓			1
History of migraine*			✓	✓		✓	3
Syncope						✓	1
Palpitations*				✓		✓	2
Neck pain*			✓	✓		✓	3
Chest pain						✓	1
Heat intolerance						✓	1
Dry mouth						✓	1
Dyspnea						✓	1
Fatigue*	✓	✓	✓	✓	✓	✓	6
Cognitive impairment*				✓		✓	2
Heart rate difference*			✓	✓		✓	3
Supine SBP						✓	1
Supine DBP						✓	1
Tilt maximum SBP						✓	1
Tilt minimum SBP						✓	1
Tilt maximum DBP	✓	✓			✓	✓	4
Tilt minimum DBP				✓		✓	2
Tilt syncope						✓	1
Previously treated pharmacologically?						✓	1

*Indicates variable was ultimately selected for the model.

### 2.4 Model creation aim 1: pharmacological therapy initiation

Variables selected by at least two of six variable selection methods were included in the model. Utilizing the selected variables, a logistic regression model was created to evaluate the odds of pharmacological initiation. As length of follow-up varied from patient to patient, observation time was forced into the regression model during variable selection and model creation. JMP Pro version 16 (SAS Institute Inc., Cary, NC, 1989–2021) was utilized for model creation.

### 2.5 Model creation aim 2: treatment changes

To maximize clinical utility of the model results and minimize redundancy, only one selected tilt table physiological measure was chosen to be included in the final model. The difference in heart rate between supine positioning and during tilt table examination was chosen. This measure was chosen three out of six times in the variable selection methods and was the most highly associated (largest effect when modeled with non-tilt covariates) and clinically relevant (direct measure of positional tachycardia) tilt table measurement. All other variables selected by at least two of the six selection methods were included in the model.

Utilizing the selected variables, a negative binomial regression model was created to examine the selected variables’ associations with the number of POTS treatment course changes. As the length of follow-up varied from patient to patient, we utilized the log of observation time as an offset term. Negative binomial regression was chosen as our regression method of choice because it is appropriate for modeling counts (in this case, number of treatment changes) in subjects while allowing for unequal follow-up times and more flexibility for the dispersion parameter (17). SAS statistical software version 9.4 (SAS Institute, Cary, NC) was utilized for model creation and diagnostics.

## 3 Results

### 3.1 Patient characteristics

A total of 322 patients were included in the study. Patient and treatment level factors can be seen in Table 3. Patients were overwhelmingly female (91%) and white (92%), with an average age of 27 at the initial visit. 26% of our population had previously been diagnosed at an outside institution prior to tilt table evaluation and diagnostic confirmation at our institution. 48% initially saw a neurologist at our institution while 52% saw a cardiologist.

**Table 3:** Patient and treatment course characteristics of full cohort: patient level factors of the full cohort, and treatment course characteristics.

Demographics and social history	
Sex, female [%, (n)]	91% (294)
Reported race, white [%, (n)]	92% (297)
Age, years [median (Q1, Q3)]	27 (22, 36.3)
Characteristics of disease and treatment courses	
Observation time years [median (Q1, Q3)]	2.08 (0.83, 3.00)
Treatment changes (unadjusted) [median (min-max)]	1 (0–12)
Department (neurology) [%, (n)]	48% (156)
Previously diagnosed (Yes) [%, (n)]	26% (85)
Antecedent event (Yes) [%, (n)]	16% (51)
Medical history [%, (n)]	
History of anxiety disorder	66% (213)
History of psychiatric disorder	75% (242)
History of autoimmune disorder	22% (71)
History of Sjogren syndrome	2% (7)
History of celiac disease	3% (11)
History Hashimoto’s thyroiditis	7% (21)
History of rheumatoid arthritis	2% (7)
History of Raynaud phenomenon	12% (39)
History of Chiari malformation	3% (10)
History of migraine	50% (161)
History of Ehlers-Danlos syndrome (EDS)	20% (64)
History of fibromyalgia	23% (73)
History of depression	49% (159)
COVID-19 infection during observation	20% (66)
Symptoms reported at initial visit (%, (n))	
Syncope	43% (137)
Palpitations	81% (262)
Neck pain	26% (84)
Chest pain	50% (160)
Dyspnea	59% (191)
Fatigue	76% (245)
Lightheadedness	79% (254)
Vertigo	80% (256)
Weakness	34% (109)
Paresthesia	48% (154)
Vasomotor symptoms	34% (110)
Sweating	48% (156)
Heat intolerance	64% (206)
Dry eyes	19% (61)
Dry mouth	26% (83)
Vision problems	43% (140)
Headache	59% (189)
Gastrointestinal symptoms	53% (171)
Cognitive impairment	38% (122)
Tilt table measurements [median (Q1, Q3) or %(n)]	
Supine systolic blood pressure (mmHg)	109 (104,117)
Supine diastolic blood pressure (mmHg)	69 (63, 75)
Tilt maximum systolic blood pressure (mmHg)	124 (115, 133)
Tilt minimum systolic blood pressure (mmHg)	107 (101, 117)
Tilt maximum diastolic blood pressure (mmHg)	82 (75, 91)
Tilt minimum diastolic blood pressure (mmHg)	68 (62, 76)
Supine heartrate (bpm)	73 (66, 81)
Tilt maximum heartrate (bpm)	117 (108, 128)
Heart rate difference (bpm)	43 (37, 50)
Tilt syncope (yes)	25% (80)

The most frequent comorbid medical conditions charted include psychiatric conditions (75%), anxiety disorders (66%), migraine (50%), depression (49%), fibromyalgia (23%), autoimmune conditions (22%), and Ehlers-Danlos syndrome (20%). 20% of patients had a confirmed COVID-19 infection during the observation period. The most reported symptoms at the initial visit include palpitations (81%), vertigo (80%), lightheadedness (79%), and fatigue (76%). All patients underwent a tilt table examination for diagnosis. The median heart rate difference from supine to head up tilt was 43 beats per minute. The median blood pressure was normotensive both before and after head up tilt. 25% of patients fainted during the tilt table examination.

### 3.2 Treatment characteristics

In total, 752 POTS-specific treatment courses were cataloged, and 429 treatment changes were observed in 322 POTS patients. A total of 130 changes (30.3%) consisted of the non-pharmacological baseline treatment to pharmacological treatment, 247 changes (57.6%) consisted of changes from one pharmacotherapy regimen to another, and 52 changes (12.1%) consisted of pharmacological discontinuation and return to the non-pharmacological baseline. Characteristics of disease and treatment courses can be seen in Table 3. The median (Q_2_) observation time was 2.08 years (Q_1_ = 0.83, Q_3_ = 3.00). Unadjusted for various observation times, individuals experienced a minimum of 0, a maximum of 12, and a median of 1 treatment changes. The therapies most used in treatment regimens included beta-blockers, purely non-pharmacological recommendations, fludrocortisone, and midodrine (Table 4). Eighty one patients out of 322 (25%) never tried pharmacotherapies and were solely treated with baseline non-pharmacological management during the observation period.

**Table 4:** Treatment uses in regimens: treatments used, the number of courses the treatment was used in, and the percentage of courses the treatment was used in.

Treatment	Number of uses	Percent utilized in all regimens
Beta-blockers	265	35.2%
Baseline non-pharmacological recommendations only	240	31.9%
Fludrocortisone	151	20.1%
Midodrine	150	19.9%
Pyridostigmine	91	12.1%
Ivabradine	71	9.4%
Prescription salt tablets	66	8.8%
Calcium channel blockers	14	1.9%
Clonidine	12	1.6%
Desmopressin	10	1.3%
Amantadine	3	0.4%
Guanfacine	3	0.4%
Droxidopa	2	0.3%
Methyldopa	2	0.3%
Venlafaxine	2	0.3%
Memantine	1	0.1%
Modafinil	1	0.1%
Propafenone	1	0.1%

Reasons for treatment changes are described in Table 5. The most cited reason for a treatment change was uncontrolled symptoms (68%) followed by side effects (23%) and other reasons (6%). Cost of medications was only cited once as a reason for treatment change. 12% of treatment changes did not contain a charted reason. Reasons for a change in treatment regimen that contained a specific drug can be seen in Table 6. The most common reason for a change in a regimen containing any one drug continued to be uncontrolled symptoms. Side effects were cited as a reason for a change most often in regimens containing pyridostigmine (41%), ivabradine (40%), prescription salt tablets (43%), or fludrocortisone (31%).

**Table 5:** Reasons given for treatment changes: reasons cited for a change in regimen for all collected treatment courses.

Reasons charted for change in regimen	Number of course changes, (%)
Uncontrolled symptoms	292 (68%)
Side effects	99 (23%)
Drug costs	1 (0.2%)
Other*	25 (6%)
Reason not charted	50 (12%)

*Other reasons: Patient decided to discontinue themselves (17), Felt no improvement (5), Developed another chronic condition that required a change (1), Worsened secondary to COVID-19 infection (1).

**Table 6:** Reasons for changes of regimens containing specific treatments: cited reasons for changes from regimens that contained a pharmacological therapy.

Regimen	Uncontrolled symptoms n (%)	Side effects n (%)	Drug costs n (%)	Other n (%)	Not charted n (%)
Baseline non-pharmacological recommendations only	121 (92%)	0 (0%)	0 (0%)	2 (2%)	8 (6%)
Contained beta-blockers*	93 (54%)	49 (28%)	1 (1%)	13 (8%)	17 (10%)
Contained Fludrocortisone*	53 (45%)	36 (31%)	0 (0%)	12 (10%)	16 (14%)
Contained Midodrine*	39 (44%)	25 (28%)	0 (0%)	7 (8%)	18 (20%)
Contained Pyridostigmine*	31 (44%)	29 (41%)	0 (0%)	5 (7%)	5 (7%)
Contained Ivabradine*	19 (42%)	18 (40%)	0 (0%)	3 (7%)	5 (11%)
Contained prescription salt tablets*	18 (43%)	15 (36%)	0 (0%)	4 (10%)	5 (12%)
Contained droxidopa*	0 (0%)	0 (0%)	0 (0%)	0 (0%)	0 (0%)
Contained desmopressin*	5 (56%)	3 (33%)	0 (0%)	0 (0%)	1 (11%)
Contained other drugs^X^	11 (34%)	14 (44%)	0 (0%)	3 (9%)	4 (13%)

*Treatment regimens changed contained one of these drugs, the drug itself is not necessarily the one removed. XDrugs include: Calcium Channel Blockers, Clonidine, Amantadine, Guanfacine, Methyldopa, Venlafaxine, Memantine, Modafinil, Propafenone.

### 3.3 Aim 1: logistic regression model

Our multivariable model adjusted for previous diagnosis, history of migraine, palpitations, fatigue, and observation time (Table 1). Results of associations with the odds of initiation of pharmacotherapy can be seen in Table 7. While controlling for other factors, the odds of starting pharmacotherapy for those who had a previous diagnosis of POTS was 2.08 times higher than those who received a diagnosis at our center (OR 2.08 [1.04–4.17] *p* = 0.04). The odds of starting pharmacotherapy for those who reported fatigue was 1.94 times higher than those who did not (1.94 [1.06–3.57] *p* = 0.03). The odds of starting pharmacotherapy for those with a history of migraines was 2.4 times higher than those who did not (OR 2.40 [1.37–4.20] *p* = 0.002). The odds of starting pharmacotherapy for those who reported palpitations was 2.62 times higher than those who did not (OR 2.62 [1.37–5.03] *p* = 0.004).

**Table 7:** Model Estimates for aim 1 pharmacotherapy initiation: each variable used in logistic regression modeling, the effect estimates for each variable in the form of odds ratios, and the respective *p*-values for each association.

Variable	Odds ratio (95% CI)	*p*-value (*α* = 0.05)
Previous diagnosis (Yes vs. No)	2.08 (1.04–4.17)	0.04
Fatigue (Yes vs. No)	1.94 (1.06–3.57)	0.03
Migraines (Yes vs. No)	2.40 (1.37–4.20)	0.002
Palpitations (Yes vs. No)	2.62 (1.37–5.03)	0.004

Observation time in days was included in the model as a control.

### 3.4 Aim 2: negative binomial regression model

Our multivariable model adjusted for history of migraine, palpitations, neck pain, fatigue, cognitive impairment, and heart rate difference (Table 2). Results of associations with number of treatment changes are summarized in Table 8. Controlling for other factors, those who report migraines at their initial visit have a 44% increase in the rate of POTS treatment changes (*p* = 0.006). Those who report palpitations at their initial visit have a 36% increase in the rate of POTS treatment changes, however this result is only marginally significant (*p* = 0.08). Those who report neck pain at their initial visit have a 27% decrease in the rate of POTS treatment changes (*p* = 0.04). Those who report fatigue at their initial visit have a 66% increase in the rate of POTS treatment changes (*p* = 0.003). Those who report cognitive impairment at their initial visit have a 28% increase in the rate of POTS treatment changes, however this result is only marginally significant (*p* = 0.06). For every 10 beats per minute increase in the change in heart rate from supine during tilt table examination there is a 13% increase in the rate of treatment changes (*p* = 0.03).

**Table 8:** Model estimates for aim 2 treatment changes: each variable used in negative binomial regression modeling, the effect estimates for each variable in the form of incident rate ratios, and the respective *p*-values for each association.

Variable	Incident rate ratio (95% CI)	*p*-value (*α* = 0.05)
History of migraine (Yes vs. No)	1.44 (1.11–1.86)	0.006
Palpitations (Yes vs. No)	1.36 (0.96–1.93)	0.08
Neck pain (Yes vs. No)	0.73 (0.54–0.98)	0.04
Fatigue (Yes vs. No)	1.66 (1.20–2.31)	0.003
Cognitive impairment (Yes vs. No)	1.28 (0.99–1.67)	0.06
Heart rate difference Δ = 10 bpm	1.13 (1.01–1.26)	0.03

## 4 Discussion

### 4.1 Patient characteristics

In this work, we described the treatment and disease courses of a sample of patients with POTS and explored the factors that may contribute to the odds of starting pharmacotherapy and undergoing changes in their management.

Our study sample was demographically like that of other studies of POTS, with the majority of patients being young white females (5, 18). The prevalence of most comorbid conditions in our sample, including migraine, fibromyalgia, autoimmune conditions, and Ehlers-Danlos syndrome, was like that of the larger population (1). However, we had a larger percentage of psychiatric and anxiety disorder diagnoses compared to previous studies (19, 20). One large cross-sectional survey study cited the number of individuals who continue to have diagnoses of a psychiatric condition after POTS diagnosis as 31%. It is important to note that the diagnoses in that study were self-reported while ours had to have been docxumented within the electronic medical record (19). Our sample size is relatively small compared to large prevalence studies and our sample may simply have a higher representation of those with mental health conditions as our institution is a tertiary care center. Additional large nationwide studies into the prevalence of true mental health disorders, with controlled diagnostic criteria, would be needed to examine these findings more thoroughly.

The most common symptoms endorsed at the initial visit in our sample were palpitations, vertigo, lightheadedness, and fatigue. These symptoms are often listed among the most common in other survey studies of POTS patients but tend to have various prevalence based on the study (19, 21). These differences are likely due to how the data is collected in each. While our collected sample reported symptoms at their initial specialist visit, others collected symptoms at later times in the disease course (19, 21).

### 4.2 Characteristics of treatment changes

We found that the most common reason for a patient to switch their treatment regimen was due to uncontrolled symptoms. This finding is not surprising given the lack of robust evidence for the efficacy of any one treatment modality (7). However, if one looks at only regimens that contained a pharmacological treatment (Table 6), the proportion of changes where side effects were listed as a reason for change ranges from 28 to 41%, depending on the drugs in the regimen. One cannot underscore enough the importance of meeting the need for high-quality trials in POTS pharmacological therapy, as the side effects of many of these medications are not trivial. Pyridostigmine, for example, can cause severe abdominal pain, diarrhea, and muscle cramps, while midodrine can cause supine hypertension and lead to severe secondary complications due to this (18). Subjecting patients to potential side effects such as these, with drugs that have little reliable evidence for efficacy, is unfortunately necessary at the current time due to the lack of response to or ability to participate in non-pharmacological management seen in some patients (7). Our finding that only 25% of our patients did not try pharmacotherapy during the observed period underscores the fact that baseline non-pharmacological recommendations are insufficient for many individuals. However, it is important to note that the number of patients able to recover without pharmacological intervention is likely much higher in the general population, as these less severe cases may be less likely to seek out specialty care.

The cost of drugs was only cited once within the chart as a reason for discontinuation of a treatment regimen. However, we cannot assess in this study whether it impacts the choice of pharmacologic management, as certain drugs with a high cost may be trialed less often in individuals with economic hardship. In addition, socioeconomic status is known to impact the patient-doctor relationship (22). Patients who cannot afford medications may not feel comfortable telling their provider, so this may be underreported in our sample.

### 4.3 Associated factor: previous diagnosis

We found that the odds of starting pharmacotherapy for those who had a previous diagnosis of POTS was 2.08 times higher than those who received a diagnosis at our center. These patients were diagnosed via outside testing and were retested at our facility. These individuals likely had suffered longer with symptoms and were potentially more severely impacted due to being referred to our specialty clinic. For these reasons, it makes logical and practical sense that they would have a higher odds of being started on pharmacotherapy and was an important factor to control for in our first model.

### 4.4 Associated factor: migraine

Migraine is one of the most common co-morbidities in POTS patients with studies reporting frequencies from 28 to 61% (23, 24). Interestingly, recent work has shown that POTS patients have sensory sensitization both with and without migraine. In addition, those with migraines without POTS had higher autonomic symptom scores and heart rate increases on tilt table examination than controls. This may suggest that migraine and POTS may act through similar and possibly overlapping pathways (23).

The odds of moving to pharmacotherapy for those with a history of migraines was 2.4 times higher than those who did not. In addition, a history of migraine was associated with a 44% increase in the rate of POTS treatment changes. There may be several explanations for these findings. Firstly, those with POTS and migraine might have a distinct pathophysiology that is more refractory to both non-pharmacological management and current therapies. It is possible that patients with migraines are used to treating their medical problems with medications. A migraine patient thus may be more likely to ask for a drug to help alleviate symptoms. One potential explanation for the increase in treatment changes is the impact of the medications used in both conditions. Fludrocortisone, a commonly used therapy in POTS is known to worsen migraine and therefore could lead to individuals seeking a change in treatment (24). The impact of other POTS treatments on migraine frequency or severity has not been extensively evaluated. Upcoming POTS clinical trials should note any medication effects on migraines given its high prevalence and potential impact on tolerability.

Propranolol, metoprolol, and timolol are often used both for migraine prevention and for heart rate control in POTS. However, when these beta blockers are taken at levels needed for migraine prophylaxis, they can lead to worsened fatigue and hypotensive episodes in POTS (24). Nebivolol has been studied for migraine prophylaxis and may carry a lower risk of causing fatigue. This drug may therefore be preferred in migraine patients with POTS, though confirmatory studies are needed (24, 25). Amitriptyline is used in migraine but may exacerbate tachycardia and fatigue in POTS. Venlafaxine is additionally used in migraines but may worsen POTS in patients with predominant hyperadrenergic symptoms. Lastly, topiramate can lead to cognitive issues which makes it a less suitable option for POTS patients with prominent cognitive symptoms (24, 25). This is additionally relevant given our finding that cognitive impairment was independently associated with an increased rate of treatment changes, though this was only marginally significant [IRR = 1.28 (0.99–1.67), *p* = 0.06]. The impacts of other migraine medications on autonomic symptoms in POTS have not yet been evaluated.

Given the prospect of a shared mechanism involving peripheral and central sensitization, therapies such as calcitonin-gene-related peptide antagonists may be promising to be effective for both conditions. This may be an exciting area for further research (23, 24). Due to the known crossover of pathophysiology, impacts of medications, and the current work showing the impact on treatment changes, POTS and migraine providers should take care in ensuring that treatment courses are ideal for the management of both conditions.

### 4.5 Associated factor: neck pain

Neck pain, most often in the form of “coat hanger pain” involving the neck and shoulders, is a well-known symptom of autonomic disorders. This phenomenon is most often described in the literature in cases of orthostatic hypotension and autonomic failure, but it has also been described in POTS (26, 27). The leading proposed mechanism of coat hanger pain in autonomic disorders is hypoperfusion of the muscles of the neck and shoulders (27). We found that those who reported neck pain at the initial visit had a 27% decrease in the incidence rate of treatment changes. One explanation for this finding is that some of the most used POTS pharmacologic therapies are those that work by increasing perfusion and raising the blood pressure such as fludrocortisone and midodrine. This suggests the possibility that patients with mainly hypoperfusion-driven symptoms may be responding better to current treatment strategies.

Patients with Ehlers-Danlos Syndrome (EDS) and POTS frequently have neck pain secondary to cervical spine hypermobility (28). Current therapies may be better at targeting pathophysiological mechanisms in this population; however, we find this less likely given that we did not find a significant association of treatment course changes with EDS.

### 4.6 Associated factor: fatigue

Fatigue is a very commonly described symptom of POTS, with some studies reporting the prevalence of fatigue as high as 91% (29). In our study, 76% of patients reported fatigue at the initial visit. The reasoning for this high incidence of fatigue in POTS is unknown. Current hypotheses suggest that this may be due to the pathology of POTS itself, the presence of comorbidities such as chronic fatigue syndrome/Myalgic Encephalomyelitis, medications used for POTS, sleep disruption, and/or the added stress of living with a chronic medical condition (29). Fatigue in POTS can contribute to the loss of employment and make non-pharmacological exercise-based disease management difficult (3, 29). The profound impact of this symptom and potential interference with not pharmacological management, may be why we found that the odds of starting pharmacotherapy for those who reported fatigue was 1.94 times higher than those who did not.

We found that the strongest factor associated with an increase in the rate of treatment changes was the presence of fatigue at the initial visit, with a 66% increase. This is not surprising given the lack of FDA-approved treatments for fatigue and the difficulty this symptom brings to completing non-pharmacological management (29). Management of fatigue in this population is only complicated by the difficulty present in identifying and quantifying it, with no gold standard or objective measurements to assess it (29). None of the current POTS therapies in use have been reliably shown to improve this disabling symptom and some medications actually worsen it. Certain beta-blockers, alpha-2 adrenergic agonists, and methyldopa all carry a risk of worsened fatigue. The use of these drugs in patients with severe fatigue may result in lower tolerability and should be avoided if possible (1, 29). Several off-label therapies for fatigue in POTS have been used including methylphenidate, atomoxetine, and modafinil, with some reports of success (30). However, one should be cautious to start POTS patients on stimulants, especially those with prominent hyperadrenergic symptoms, as they are known to cause tachycardia and their use has not been highly studied in this context (1).

Given the frequency, lack of evidence-based therapies, and the association with frequent therapy changes, fatigue should be a primary target and outcome of studies evaluating treatments for POTS. More work needs to be done to create objective measurements of fatigue for studies and establish consistent use of subjective fatigue questionnaires. POTS clinicians should incorporate counseling on the non-pharmacological management of fatigue within standard practice (29). Finally, the link between POTS and conditions such as chronic fatigue syndrome/Myalgic Encephalomyelitis needs to continue to be investigated especially the impact of exercise on the overlapping population, which can worsen symptoms in a subset of patients (31).

### 4.7 Associated factor: palpitations

We found that the odds of starting pharmacotherapy for those who reported palpitations was 2.62 times higher than those who did not. Palpitations are a commonly reported symptom and can be quite distressing (19, 21). Reduction of palpitations likely represent a large reason people seek pharmacological therapy as readily available drugs such as beta blockers can be affective in minimizing them (9). Although it did not reach statistical significance (*p* = 0.08), in model two we found that those who report palpitations at their initial visit have a 36% increase in the rate of POTS treatment changes. This could indicate that palpitations continue to be a troublesome symptom for patients even after pharmacotherapy has been tried, leading to a higher rate of changes.

### 4.8 Associated factor: heartrate increase on tilt table examination

Attempts have been made in the literature to separate POTS into clinical “subtypes” including hyperadrenergic, neuropathic, and hypovolemic. However, there has not been a consensus on criteria for subtypes, and most patients have overlapping features. Some clinicians, including those at our institution, instead use these phenotypes and proposed features to conceptualize the use of certain off-label therapies based on mechanisms (5). Although these subtypes are not fixed, it has been thought that those with more prominent hyperadrenergic features have a more exaggerated heart rate response to head-up tilt (24). It has been shown in children that higher heart rate differences at 5 and 10 min of tilt are associated with a poorer prognosis of symptomatic recovery after 3 months of treatment (32).

In our study we found that for every 10 beats per minute increase in the heart rate difference in tilt table testing, there is a 13% increase in the rate of treatment changes. Our findings in the context of existing literature may indicate that current treatment strategies are insufficient for those with prominent and exaggerated heart rate increases. However, this requires more study. It is important to note that there is evidence of diurnal variability in POTS in that heart rate differences, other hemodynamic parameters, and symptoms may be more pronounced in the morning than in the afternoon (24, 33). We did not control for the time of day in this study. This may affect the true utility of our heart rate findings and thus they should be interpreted with caution.

### 4.9 Limitations and future directions

The ideal study evaluating POTS treatment would frame success in a more direct manner, rather than through change, as success is the ultimate goal. However, there are currently no POTS-specific scales, biomarkers, or questionnaires that can capture POTS treatment success accurately and consistently (8). By observing treatment changes in aim 2, we were able to clearly measure patient outcomes given the lack of defined “success” in the literature. Future work in the field should focus on the development of POTS-specific outcome measures (8). Our study reflects the treatment practices at one tertiary care institution. Prescribing practices may differ substantially at other institutions and thus this is a possible limitation in the generalizability of the results of both aims. Sex was selected as a factor in only one variable selection technique. Although we do not believe sex is a significant contributor to treatment changes, future pharmacological studies should continue to monitor for sex specific responses to treatment.

In this study, only medications taken for the purpose of symptom management of POTS were recorded. Medications taken for comorbidities were not taken into account. Therefore, it is unknown whether the management of POTS was impacted based on medication use for other comorbidities. Our institution recommends the same baseline non-pharmacological management for POTS to all patients, however data concerning the compliance with these recommendations was not available to us. Future studies should incorporate patient surveys and standardization of charting specific non-pharmacological management strategies utilized by each patient. Randomized clinical trials should strive to include data on reported use of non-pharmacological strategies such as compression stockings, dietary changes, hydration and exercise regimens as specific management may modify the effectiveness of pharmacological treatment strategies.

## 5 Conclusion

The purpose of this work was to explore whether there were factors associated with the odds of starting pharmacological therapy in POTS and with repeated changes in POTS treatment strategies at our tertiary care center. In aim 1, we found that a history of migraine headaches, reported fatigue, reported palpitations and a previous POTS diagnosis at an outside institution were found to be associated with a higher odds of starting pharmacotherapy for POTS symptoms. In aim 2, we found that a history of migraine headaches, reported fatigue, and higher heart rate differences on tilt table examination were found to be associated with an increase in the rate of POTS treatment changes, while reported neck pain was associated with a decrease in the rate. Furthermore, we discussed how the identification of these factors could guide current care practices, and impact future work in the field. We hope that this work can inspire others to include considerations of the factors we found that may represent groups of patients with the highest need for novel, evidence-based therapies.

## Data availability statement

The datasets presented in this article are not readily available because the current dataset is being utilized in ongoing retrospective research and thus the data will be kept within the institution at this time. Requests to access the datasets should be directed to RW, WILSONR3@ccf.org.

## Ethics statement

The studies involving humans were approved by the Cleveland Clinic Institutional Review Board. The studies were conducted in accordance with the local legislation and institutional requirements. The ethics committee/institutional review board waived the requirement of written informed consent for participation from the participants or the participants’ legal guardians/next of kin because the project was a retrospective chart review and posed minimal risk.

## Author contributions

ST: Conceptualization, Data curation, Formal analysis, Investigation, Methodology, Validation, Visualization, Writing – original draft, Writing – review & editing. RZ: Data curation, Formal analysis, Resources, Writing – review & editing. CC: Conceptualization, Methodology, Validation, Writing – review & editing. AN: Conceptualization, Data curation, Formal analysis, Methodology, Resources, Supervision, Validation, Visualization, Writing – review & editing. TS: Conceptualization, Methodology, Supervision, Validation, Writing – review & editing. RW: Conceptualization, Data curation, Investigation, Methodology, Project administration, Resources, Supervision, Validation, Writing – review & editing.
