# Supplementary material for: Factors associated with initiation of pharmacological therapy and treatment changes in postural orthostatic tachycardia syndrome
Source: Front Neurol. 2024 Jun 20;15:1411960. doi: 10.3389/fneur.2024.1411960 (PMC11222644; doi:10.3389/fneur.2024.1411960)
Supplement: Supplementary file 1 [file Table_1.docx]

**Supplementary Table 1: Variables and their definitions**

*Variables eligible for entry into variable selection methods and their definitions.*

| Variable | Definition |
| --- | --- |
| Treatment change | Treatment change or discontinuation of a treatment regimen specifically for POTS after an initial visit with a POTS specialist at our institution. |
| Observation Time | Time from date of initial visit to date of last note confirming POTS treatment regimen. |
| Sex | Reported biological sex at the initial visit with a POTS specialist at our institution. |
| Age | Reported age (years) at the initial visit with a POTS specialist at our institution. |
| Race | “White” or “Other” self-reported |
| Department | Department of initial evaluation, Neurology or Cardiology |
| Previously diagnosed at outside hospital? | Whether the patient had been previously diagnosed at another institution. |
| Previously treated pharmacologically? | Prior to tilt table diagnosis at our institution, were they treated for POTS with pharmacological management? |
| Antecedent event? | Did the patient experience a health event prior to symptom onset (viral infection, TBI, pregnancy etc.) |
| History of Anxiety Disorder | Confirmed diagnosis of a DSM-IV anxiety disorder defined by a qualifying diagnosis listed or discussed in two separate notes. |
| History of Psychiatric Disorder | Confirmed diagnosis of a DSM-IV psychiatric disorder defined by a qualifying diagnosis listed or discussed in two separate notes. |
| History of Migraine | Medical history of migraine documented at initial visit |
| History of an Autoimmune Condition | Medical history of an autoimmune condition documented at initial visit |
| History of Ehlers-Danlos syndrome (EDS) | Medical history of EDS documented at initial visit |
| History of Fibromyalgia | Medical history of fibromyalgia documented at initial visit |
| History of Depression | Medical history of depression documented at initial visit |
| Syncope | Symptom present at the initial visit, yes or no |
| Palpitations | Symptom present at the initial visit, yes or no |
| Neck pain | Symptom present at the initial visit, yes or no |
| Chest pain | Symptom present at the initial visit, yes or no |
| Dyspnea | Symptom present at the initial visit, yes or no |
| Fatigue | Symptom present at the initial visit, yes or no |
| Lightheadedness | Symptom present at the initial visit, yes or no |
| Vertigo | Symptom present at the initial visit, yes or no |
| Weakness | Symptom present at the initial visit, yes or no |
| Paresthesia | Symptom present at the initial visit, yes or no |
| Vasomotor Symptoms | Symptom present at the initial visit, yes or no |
| Sweating | Symptom present at the initial visit, yes or no |
| Heat intolerance | Symptom present at the initial visit, yes or no |
| Dry eyes | Symptom present at the initial visit, yes or no |
| Dry mouth | Symptom present at the initial visit, yes or no |
| Vision problems | Symptom present at the initial visit, yes or no |
| Headache | Symptom present at the initial visit, yes or no |
| Gastrointestinal symptoms | Symptom present at the initial visit, yes or no |
| Cognitive Impairment | Symptom present at the initial visit, yes or no |
| Supine Systolic Blood Pressure (SBP) | Average supine systolic blood pressure prior to tilt table examination. |
| Supine Diastolic Blood Pressure (DBP) | Average supine diastolic blood pressure prior to tilt table examination. |
| Tilt Maximum Systolic Blood Pressure (SBP) | Maximum systolic blood pressure in the first ten minutes of head up tilt. |
| Tilt Minimum Systolic Blood Pressure (SBP) | Minimum systolic blood pressure in the first ten minutes of head up tilt. |
| Tilt Maximum Diastolic Blood Pressure (DBP) | Maximum diastolic blood pressure in the first ten minutes of head up tilt. |
| Tilt Minimum Diastolic Blood Pressure (DBP) | Minimum diastolic blood pressure in the first ten minutes of head up tilt. |
| Supine Heartrate (HR) | Average supine heart rate prior to tilt table examination. |
| Tilt Maximum Heartrate (HR) | Maximum heart rate within the first ten minutes of head up tilt |
| Heart Rate Difference | Difference between the average supine heart rare and the maximum heart rate during the first ten minutes of head up tilt |
| Tilt Syncope | Did the patient experience syncope during the tilt table examination? |
| Echocardiogram completed | Echocardiogram documented yes or no |
| COVID 19 infection during observed period? | PCR confirmed COVID-19 during the observed time period. |
